# Musical Bruits and Accidents to Workmen

**Published:** 1908-03-21

**Authors:** 


### Medico-Legal Points.

### MUSICAL BRUITS AND ACCIDENTS TO WORKMEN.

The question of traumatic rupture of an aortic valve assumes quite a new importance nowadays in connection with the Workmen's Compensation Act, and the other Acts allied to it. The following is a case in point:—

A man aged 42, employed as a mechanic, applied one day for a certificate to say that he was suffering from the effects of an accident incurred during his work. His main symptom was dyspnœa. He gave the history that, having always been in perfectly good health hitherto, he had been engaged some weeks previouly in repairing a flywheel weighing about a hundredweight. Suddenly the supports of this flywheel gave way, and it fell, striking him forcibly upon the arms and the front of the chest. Instantaneously during the violent muscular effort he made to resist the blow and to prevent himself from being altogether crushed, he felt something break with a snap within his thorax, and at the same time he experienced a very acute and severe pain behind his sternum. No bone was broken, no marked evidence of bruising appeared, the man's extended arms having apparently broken the impetus of the wheel, and prevented any great external injury to the chest wall. Nevertheless, from the moment of the accident, the man was continuously afflicted with severe dyspnœa upon the least exertion; it was impossible for him to do any work at all. He was therefore claiming compensation, for complete disablement, from his employers.

Upon examination he was found to have a loud diastolic aortic murmur. The heart was not enlarged, and the pulse was not typically water-hammer in type, but it was clear that the patient had aortic regurgitation. There was no mitral bruit. The aortic murmur differed from the ordinary soft-blowing bruit that usually replaces the second sound; it was very loud, widely transmitted, and it had a so-called "musical" character resembling rather the mewing of a cat than ordinary music. The heart's action was regular, and not very rapid. The patient's other organs were apparently sound; and there was no evidence of syphilis. He affirmed that he had suffered from no dyspnœa before the accident, and that he had never had acute rheumatism nor any other serious illness.

The question arose as to whether the condition was to be regarded as one of traumatic rupture of the aortic valve, or whether it might not be that the injury was but a pretext for claiming compensation when the patient might really have been ill before the accident occurred. The answer must to a large extent remain one of opinion; that is to say, it must rest upon analogy with other cases of a similar kind. It is quite obvious that, to resolve the difficulty completely, it would be necessary to know whether there had been any aortic bruit to be heard before the accident. In the present case this was not known. The interpretation therefore rested ultimately upon the significance of the musical character of the bruit.

In the first place, the majority of cases of a similar kind, in which before the days of these important Acts of Parliament the valvular lesion was thought by physicians to be essentially traumatic, the bruit was particularly noted as being loud and musical.

In the second place, almost all authorities regard the existence of a musical bruit as evidence of gross lesions in connection with the valve flaps themselves. It is met with particularly in cases of ruptured chordæ tendineæ, and in cases of perforations and ulcerations of the valves. It is therefore frequently observed in conditions of malignant or fungating endocarditis, which probably cause greater destructive lesions of the valves than does any other condition. It seems likely, therefore, that traumatic affections of the valves, comparable in the splits and tears they cause to the different varieties of spontaneous rupture or perforation of the valves, would be accompanied by the same physical signs, particularly the musical character of the bruit, as are produced by an acute fungating endocarditis. It is also clear, of course, that a valve already diseased would be more likely to rupture as the result of a sudden muscular effort, or of a direct blow to the chest, than would a perfectly healthy valve. It is impossible, in a case such as the one quoted, to be quite sure that the aortic valve was moderately healthy before the accident. The question is: to what extent was the accident itself responsible for the incapacity of the man to continue at work? How long would he have been likely to be able to go on as a mechanic if he had not met with this accident? There was no evidence whatever of fungating endocarditis in the case; there was no proof that the man had had syphilis as a likely cause for pre-existing aortic disease; he was not addicted to the free use of alcohol. The symptoms came on for the first time immediately after the accident; the heart was not enlarged, so that there was no evidence of aortic regurgitation of long standing. It seemed clear that the injury was the direct cause of the man's incapacity for further work, and that it had ruptured one of the aortic flaps.

The importance of the case lies in the significance that can be attached to the musical character of the bruit. It seems that it is possible to have at least a strong suspicion that the valvular lesion is traumatic, if the bruit has a loud musical character, if the patient has presented no symptoms of heart disease previous to the accident, if the heart shows no signs of an old aortic lesion, and if the injury and the muscular effort were such as have been known to cause sudden rupture of the aortic valve, with loud musical bruit, in former cases.

Traumatic rupture of an aortic valve is a lesion which very seldom becomes compensated properly, even for ordinary existence, still less for being able to work. Hence, in a case such as the above, if the interpretation given to it upon medical grounds be the correct one, compensation for complete disablement would be due. It is obvious, therefore, that from the employers' point of view there may sometimes be a very great significance in the fact that a particular aortic bruit is musical.

